# Symptoms and level of functioning related to comorbidity in children and adolescents with ADHD: a cross-sectional registry study

**DOI:** 10.1186/s13034-020-00336-4

**Published:** 2020-07-29

**Authors:** Marie Elwin, Tove Elvin, Jan-Olov Larsson

**Affiliations:** 1 University Health Care Research Center, Region Örebro County, Örebro, Sweden; 2 Karolinska Institutet, Stockholm, Sweden; 3 Faculty of Medicine and Health, Örebro University, Örebro, Sweden

**Keywords:** Attention deficit hyperactivity disorder, Children, Adolescents, Comorbidities, Global functioning, Symptom assessment

## Abstract

**Background:**

It is well known that a wide range of psychiatric disorders co-occur with attention deficit hyperactivity disorder. In this study we aimed to examine the associations of psychiatric comorbidity in ADHD with symptom severity and level of functioning.

**Methods:**

We used data from the Swedish National Quality Registry for ADHD Treatment Follow-up and identified comorbid diagnoses in a sample of 3246 Swedish children and adolescents with ADHD. We investigated the association of comorbidity with symptom severity and level of function by multiple linear regressions.

**Results:**

Autism spectrum disorder, anxiety and affective disorders, oppositional defiant disorder or conduct disorder, learning disorders, and multiple comorbid disorders associate to lower levels of functioning compared to ADHD only. Multiple comorbidity, autism spectrum disorder, oppositional defiant or conduct disorders and tic disorders relate to ADHD symptom severity.

**Conclusions:**

Comorbidity subgroups with ADHD differ in functional impairment and ADHD symptoms severity. Information on comorbidity profiles could be used for treatment planning more adapted to the individual. Especially those who have autism spectrum disorders and multiple comorbid disorders are at risk of severe ADHD symptoms and low level of functioning.

## Background

Attention deficit hyperactivity disorder (ADHD) is a neurodevelopmental disorder characterized by symptoms of inattention and/or hyperactivity and impulsivity causing impairment in two or more settings. DSM-5 classifies three subtypes: inattentive presentation, hyperactive-impulsive presentation, and combined presentation. For a diagnosis of ADHD, symptoms should have manifested before 12 years of age and persisted for a minimum of 6 months [1]. ADHD is usually diagnosed in childhood with a prevalence of 3–6% in children and adolescents and about 2.5% in adults. ADHD often persists into adulthood in one-half to two-thirds of those diagnosed in childhood [2].

ADHD is associated with an increased risk of having other coexisting psychiatric disorders, both in childhood and in adulthood [3]. Comorbidity with neurodevelopmental and psychiatric disorders is common in both child and adult ADHD: in clinically referred children the rate is approximately 65% to 85% [4]. Comorbidity rates are smaller in population-based samples [5–7].

Common childhood ADHD comorbidities are oppositional defiant disorder (ODD) and conduct disorder (CD) [8], learning disorders [9], autism spectrum disorders (ASD) [10], anxiety and affective disorders, and tic disorders [11–13]. Comorbidity with affective and anxiety disorders is the same in adulthood, in addition to substance use disorders and personality disorders co-occurring in adulthood [3, 14, 15].

Research indicates that patients with serious mental disorders have comorbidity as a common factor [16]. Comorbid disorders in patients with ADHD are also a risk factor for the persistence of ADHD into adulthood [17, 18]. A review and meta-analysis showed comorbid CD, affective disorder, and ADHD severity to be major factors for persistence of ADHD from childhood to adulthood [19]. In a study by Arnold et al. [17], children with comorbid internalizing disorders (anxiety and depression) were more likely to have ADHD persisting into adulthood compared to children with ADHD and externalizing comorbidity (ODD/CD), children with pure ADHD, and even children with severe childhood ADHD.

We do not fully understand the clinical impact of comorbidity in ADHD. The present study will provide an overview of common comorbid diagnoses and comorbid combinations in a large sample of children and adolescents with ADHD, who have contact with psychiatric services in Sweden. We assessed the association of comorbidity with clinical ratings of global psychosocial functioning. In addition the association of comorbidity with severity of ADHD symptoms was assessed. We hypothesized lower global psychosocial function in individuals with comorbidities, especially multiple comorbidities. We also hypothesized that ODD/CD is related to the dimensions of ADHD and that it results in more severe ADHD symptoms than in individuals with ADHD only.

## Methods

### Participants

Participants in the study were identified through the Swedish National Quality Registry for ADHD Treatment Follow-up (BUSA). BUSA is an internet-based quality assurance register whose target population is all Swedish individuals with a confirmed ADHD diagnosis according to ICD-10 (F90.0–F90.8). However, BUSA is a non-mandatory registry and patients can decline participation. Thus, only a subset of individuals with ADHD is included. The registry holds individualized information on demographics, comorbid diagnoses, pharmacotherapy and other treatment interventions, and ratings of symptoms by clinicians, parents, teachers, and the individuals with ADHD themselves [20]. All patients had received a clinical diagnosis of ADHD according to ICD-10 and DSM-IV. DSM-5 was published in 2013 but it was not fully implemented in Swedish psychiatry during most of the study period, however, the exclusion criterion of ASD for ADHD diagnosis according to DSM-IV was often disregarded. Consequently many patients have a concurrent diagnosis of ADHD and ASD allowed for in the revised criteria in DSM-5. Both the ADHD and the comorbid diagnoses had been assessed by clinicians in psychiatric units across Sweden. Comorbid diagnoses were registered according to the ICD-10. All diagnoses were registered in the patients’ medical records and were entered into the BUSA registry by personnel in 40 psychiatric units that report to BUSA.

**Fig. 1:**
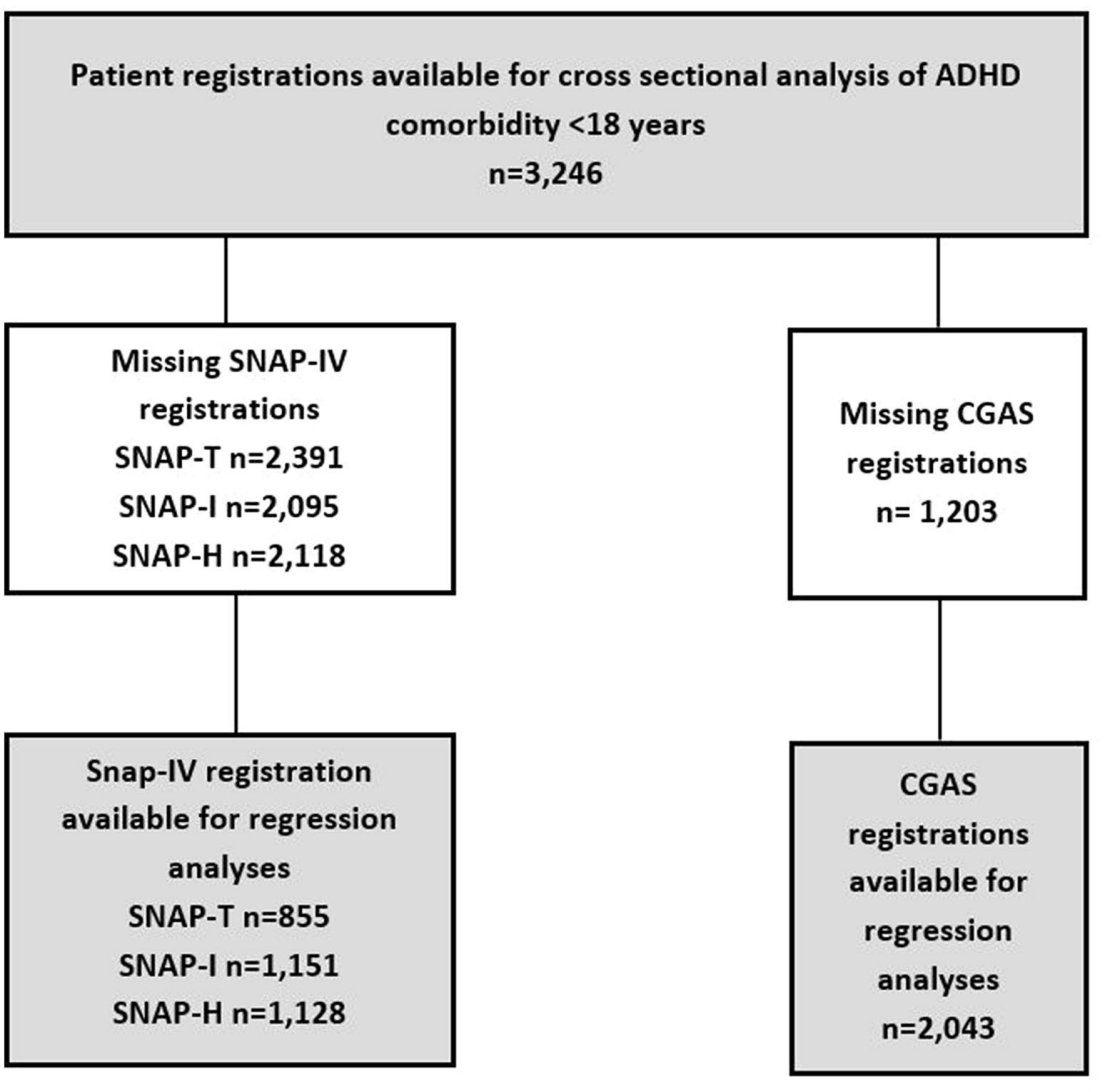
Flowchart of patients with ADHD diagnoses registered in BUSA during 2016–2017. (*CGAS* Children’s Global Assessment Scale, *SNAP-IV* Swanson, Nolan, and Pelham Questionnaire. The SNAP-IV total scale, inattention and hyperactivity/impulsivity subscales are parent ratings

BUSA offers an opportunity to study comorbidity in a large group of individuals with ADHD diagnoses, although this is not the primary aim of the registry. We analyzed data from the revised latest version of BUSA (applied from 2016), consisting of registrations from the period 14 January 2016 to 20 December 2017. All registered individuals under 18 years of age were included in the study (Fig. 1).

### Procedure and measures in the study

Before the analyses, we searched the data files for duplicates and errors in the data, and we coded categorical variables. Descriptive characteristics from BUSA used in the study were gender, age, type of ADHD, and comorbid diagnoses. We included in the analyses common childhood ADHD comorbidities referred to above and commonly occurring psychiatric disorders according to recent European and Swedish registry based data: learning disorders ODD/CD, anxiety disorders, affective disorders, tic disorders, and autism spectrum disorders [4, 7].

No patient in this sample was diagnosed with bipolar disorder. Patients diagnosed with substance abuse or personality disorders were less than .5%. Because of the rarity of these disorders, most certainly due to the young age of the patients in our sample, we did not include these disorders in our analyses.

The level of global psychosocial functioning was assessed with the Children’s Global Assessment Scale (CGAS) [21], and severity of ADHD symptoms was assessed with the Swanson, Nolan, and Pelham Questionnaire (SNAP-IV) [22].

The CGAS is a clinician-rated scale of global functioning in children and adolescents, it measures global functioning as a single factor. The CGAS can be used to rate the level of global functioning in children and adolescents from 4 to 20 years of age. The scale ranges between 1 and 100, with 1 representing the lowest level of functioning. A clinician rates the level of global functioning during the past month, at home, in school, and with friends. The scale is divided into 10-point intervals with a verbal description for each interval. The psychometric properties of the CGAS show moderate reliability [23]. The CGAS is widely used clinically and in research, and some studies have used it as an outcome measure [24].

SNAP-IV is a parent- or teacher-report screening of ADHD symptoms in children and adolescents. In this study, parent ratings were used. It rates the severity of current symptoms on a scale from 0 to 3. Higher scores indicate greater levels of ADHD symptoms. The Swedish version used in BUSA comprises 30 questions. Items 1–9 correspond to inattention criteria in DSM-IV. Items 11–19 correspond to the criteria for hyperactivity and impulsivity, and items 21–28 are questions on ODD. Item 10 is a summary score for attention deficit, and item 20 is a summary score for hyperactivity and impulsivity. Item 29 is a DSM-III-related item on ODD, and item 30 is a summary score for ODD. Test–retest reliability for SNAP-IV is evaluated as .66–.92 [22], and internal consistency is .94 (parent rating) and .97 (teacher rating) [25]. Inter-rater reliability is judged to be moderate. Factor analysis of SNAP-IV has supported the hypothesis of attention deficit and hyperactivity/impulsivity as separate dimensions, with a strong general ADHD factor [26].

Some patients in the registry had incomplete data. Some types of data, for example, scale measurements, were missing. Analyses of missing data showed no differences between those with CGAS data (n = 2043) and those without (n = 1203) in age (χ^2^ = .556, *p* = .46) or in gender (χ^2^ = 3.27, *p* = .70). There was less comorbidity (χ^2^ = 4.55, *p* = .03), in the group with missing CGAS data. For SNAP -Total (2391 with missing data and 855 with data), there were no differences in gender (χ^2^ = 1.21, *p* = .27) or age group, χ^2^ = .18, *p* = .67). The missing-data group had less registered comorbidity (χ^2^ = 80.15, *p* = < 001).

### Statistical analyses

We identified comorbidity and combinations of comorbidity in the dataset. Judged from histograms and the values for skewness and kurtosis, the distribution of CGAS and SNAP-IV total scale score were approximately normal. (For CGAS, skewness = .593, kurtosis = .570; for SNAP-total score, skewness = .032, kurtosis = − .556.) We tested the assumptions for linear regression, that is, linearity, normality of residuals, and equality of variances. The P plots of standardized residuals showed normally distributed residuals with roughly equal variance. We analyzed the influence of comorbidity on CGAS scores, on SNAP-IV total scores, and on SNAP-IV subscale scores; inattention and hyperactivity/impulsivity. All associations were assessed by multiple linear regressions. Comorbid diagnoses, gender, and age were independent variables. Overall functioning (CGAS) and ADHD symptom severity (SNAP-IV total score, inattention and hyperactivity/impulsivity subscale scores) were dependent variables in separate analyses. We ran the analyses in IBM SPSS Statistics for Windows, version 22.0 (IBM Corp., Armonk, NY, USA).

## Results

Of the 3246 included participants, the majority, 65.2%, (25.7% females) were diagnosed with combined ADHD (mean age 12.14 ± 2.86 years), followed by 18.4% with the inattentive type (34.2% females, mean age 13.71 ± 2.37 years), the hyperactive/impulsive type 4.4%, (20.8% females, mean age 12.10 ± 2.98 years) and 3.4% were diagnosed with ADHD not otherwise specified (32.7% females, mean age 12.24 ± 3.01 years). The proportion of missing data on ADHD subtype was 8.6%. Approximately 38% of the total sample were female. The largest group 51.8% were in the age range 13–17 years, the mean for the total group was 12.5 ± 2.9 years (range: 4–17 years).

Table 1 presents the frequencies of comorbidity together with their descriptive values in CGAS and SNAP-IV total scale.

**Table 1:** Comorbid patterns and related CGAS and SNAP-IV total scale statistics

Diagnostic categories	Diagnostic categories n/percentage	CGAS Mean (SD) Min–max	SNAP-IV- total scale Mean (SD) min–max
ADHD No comorbidity	1802/55.51	62.39 (10.65) 36–95 n = 1105	1.45 (.63) .10–3.0 n = 363
ADHD + ASD	779/24	54.95 (9.93) 25–92 n = 445	1.57 (.61) .10–3.0 n = 241
ADHD + anxiety	120/3.70	55.96 (8.24) 32–79 n = 95	1.48 (.64) .10–2.70 n = 42
ADHD + ODD/CD	116/3.57	52.18 (7.80) 31–72 n = 92	1.81 (.62) .10–3.0 n = 49
ADHD + learning disorders	102/3.14	60.42 (9.93) 40–85 n = 57	1.23 (.64) .20–2.50 n = 21
ADHD + tic disorder	49/1.51	60.24 (8.70) 41–80 n = 33	1.69 (.50) .40–2.30 n = 14
ADHD + affective disorders	29/(.89)	54.25 (6.96) 44–69 n = 24	1.43 (.66) .40–2.80 n = 12
Multiple comorbidity	249/7.7	52.45 (8.63) 30–88 n = 192	1.71 (.59) .50–3.0 n = 113
Total	3246/100	58.89 (10.80) 25–95 n = 2043	1.54 (.63) .10–3.0 n = 855

*CGAS* the Children’s Global Assessment Scale, *SNAP-IV* the Swanson, Nolan, and Pelham Questionnaire. ADHD: F90.0, F90.1, F90.8, F90.9, F98.8, F90.0A, F90.0B, F90.0C, F90.X; ASD: F84.0, F84.1, F84.5, F84.9; anxiety: F41.0–F41.3, F41.8–F41.9, F43.0–F43.2, F43.8W, F43.9; ODD/CD: F91.0–F91.3, F91.8–F91.9; learning disorders: F81.0–F81.3, F81.8–F81.9; tic disorders: F95.0–F95.2, F95.8, F95.9; affective disorders: F33.0–F33.4, F32.0–F32.2, F32.8–F32.9

In the total sample, 44.5% had one or more of the six diagnoses that were investigated. Table 1 shows all combinations and an aggregated category of multiple comorbidity. Disregarding combinations with other diagnoses, the frequencies for the separate diagnoses were as follows: ASD 29.4%, anxiety disorders 7.4%, affective disorders 2.9%, ODD/CD 5.5%, tic disorders 3.3%, and learning disorders 4.4%. The most frequent multiple comorbid combinations were ASD with anxiety disorders, ASD with tic disorders, ASD with ODD/CD, and anxiety disorders with affective disorders. A Chi squared test comparing frequencies of single comorbidities in boys and girls showed a significant interaction effect for ASD, with boys more likely to have ASD (χ^2^ = 14.84, *p* = < .001) and tic disorders (χ^2^ = 18.82, *p* < .001). Girls were more likely to have anxiety disorders (χ^2^ = 92.19, *p* < .001), affective disorders (χ^2^ = 14.28*, p* < .001), and multiple comorbidity (χ^2^ = 4.69, *p* = .030). There were no gender differences in learning disorders or ODD/CD. A chi-squared test comparing frequencies of single comorbidity in a younger group 4–12 years, and an older group 13–17 years showed a significant interaction effect for ASD and age: the younger group was more likely to have ASD (χ^2^ = 6.46, *p* = .011) and ODD/CD (χ^2^ = 32.54, *p* < .001). The older group was more likely to have anxiety disorders (χ^2^ = 30.74, *p* < .001), affective disorders (χ^2^ = 11.20, *p* < .001), and multiple comorbid disorders, (χ^2^ = 29.14, *p* < .001). There were no associations between age and learning disorders or between age and tic disorders.

### Regression analyses

CGAS was regressed on the comorbid disorders and demographic variables gender and age, with no comorbid disorder as reference (Table 2).

**Table 2:** Multiple linear regression: comorbid diagnoses as predictors of CGAS total score among children and adolescents

	B (95% CI)	SE B	β	*P*
Constant	52.11 (50.02 to 54.20)	1.06		< .001
Autism spectrum disorder	− 7.54 (− 8.61 to to 6.47)	.55	− .29	< .001
Anxiety disorders	− 8.52 (− 10.61 to − 6.43)	1.06	− .17	< .001
ODD/CD	− 8.87 (− 10 to − 6.80)	1.06	− 17	< .001
Learning disorders	− 2.99 (− 5.58 to − .40)	1.32	− .05	.023
Tic disorders	− 2–17 (− 5.54 to 1.20)	1.72	− .03	.21
Affective disorders	− 10.29 (− 14.25 to − 6.34)	2.02	− 10	< .001
Multiple disorders	− 10.99 (− 12.49 to − 9.50)	.76	− .30	< .001
Age at registration	.88 (.73 to 1.03)	.08	.24	< .001
Gender	− .70 (− 1.67 to .26)	.49	− .03	.15

CGAS, the Children’s Global Assessment Scale; Adjusted *R*^*2*^, .194; B, unstandardized coefficient; β, standardized beta coefficient; CI, confidence interval; SE, standard error; ODD, oppositional defiant disorder; CD, conduct disorder

Gender had no effect on CGAS scores, but age was significantly and positively associated with higher CGAS. On average, CGAS scores were .88 points higher for every year of age, or 8.8 points for 10 years, thus the older patients had less functional impairment.

All comorbid disorders except tic disorders were significantly and negatively associated with CGAS. Children and adolescents with these disorders had from (on average) − 10.99 (multiple comorbidity) to − 2.99 (learning disorders) CGAS scores compared to children with ADHD alone.

We also investigated whether comorbidity was associated with severity of ADHD symptoms, as measured by the SNAP-IV subscales: total (T), inattention (I), and hyperactivity/impulsivity (H). The independent variables were the same as in the first regression analysis (Table 3).

**Table 3:** Multiple linear regression: comorbid diagnoses, gender and age as predictors of ADHD symptoms among children and adolescents

	SNAP-T n = 855			SNAP-I n = 1151			SNAP-H n = 1128		
	B (95% CI)	SE B	*p*	B (95% CI)	SE B	*p*	B (95% CI)	SE B	*p*
Constant	2.29 (2.09–2.50)	.105	< .001	2.18 (1.99–2.36)	.09	< .001	2.57 (2.35–2.78)	11	< .001
ASD	.13 (.03–.23)	.05	.009	.21 (.13–.30)	.04	< .001	.12 (.03–.22)	.05	.013
Anxiety	.18 (− .02 to .37)	.10	.076	.17 (− .04 to .37)	.10	.107	.24 (− .01 to .48)	.12	.055
ODD/CD	.27 (.10–.45)	.09	.003	.07 (− .12 to .26)	.10	.465	.16 (− .06 to .38)	.11	.151
Learning disorder	− .18 (− .44 to .09)	.13	.186	− .21 (− .45 to .02)	.12	.078	− .16 (− .44 to .12)	.14	.254
Tic disorder	.35 (.03–.67)	.16	.030	.11 (− .18 to .40)	.15	.447	− .11 (− .44 to .22)	.17	.512
Affective disorder	.08 (− .26 to .42)	.17	.635	.11 (− .22 to .44)	.17	.506	.01 (− .37 to .44)	.20	.943
Multiple disorders	.35 (.22–.48)	.06	< .000	.35 (.22–.48)	.07	< .001	.34 (.19–.49)	.08	< .001
Age (years)	− .07 (− .08 to − .05)	.01	< .001	− .04 (− .05 to − 02)	.01	< .001	− .10 (− .12 to − .08)	.01	< .001
Gender	− .02 (− .11 to .07)	.05	.653	− .09 (− .17 to − .01)	.04	.031	.01 (− .09 to .11)	.05	.880
	Adj. R^2^ = 114			Adj. R^2^ = .057			Adj.R^2^ = .135		

*SNAP-IV* the Swanson, Nolan, and Pelham Questionnaire, *SNAP-T* total scale, *SNAP-I* inattention subscale, *SNAP-H* hyperactivity/impulsivity subscale, *B* unstandardized coefficient, *CI* confidence interval, *SE* standard error, *β* standardized beta coefficient, *ASD* autism spectrum disorder, *ODD* oppositional defiant disorder, *CD* conduct disorder

The effect of gender on SNAP-IV inattention subscale score was significant (boys had on average higher scores of inattention than girls). Age was associated negatively with all ADHD scales, (total scale, inattentive and hyperactivity/impulsivity subscales), demonstrating more severe ADHD symptoms among the younger patients.

Multiple comorbidity and ASD were positively associated with all subscale scores of SNAP-IV, meaning more inattention and hyperactivity/impulsivity symptoms in the presence of multiple comorbidity and ASD. ODD/CD and tic disorders were positively associated with SNAP-IV total scale.

## Discussion

We aimed to examine the associations of comorbidity with global level of functioning and with severity of ADHD symptoms. The results for the influence of comorbidity on global functioning confirm our hypothesis that comorbidity is associated to reduced functioning, this align with prior research that showed a strong relation of comorbidity to low functioning e.g. work disability or substantial limitation in functioning due to comorbidity of mental disorders [16]. Those with a lower symptom burden (only ADHD) have better global functioning which is not surprising. The types of symptoms represented in the different comorbid disorders have various impacts on the ability to function. ASD is most closely linked to impaired psychosocial function, learning and tic disorders having low or no impact respectively. The persistent difficulties in social interaction and communication in ASD in combination with ADHD characteristics of distractibility and impulsiveness may lead to more pronounced social impairments explaining the decrease in CGAS scores. Our hypothesis that having multiple comorbidities is related to low global functioning was confirmed.

The results revealed that the presence of ASD, multiple comorbidities ODD/CD and tic disorders is also associated with more ADHD symptoms in this sample of children and adolescents with ADHD. Similar finding of significantly higher SNAP-IV total scores (parent ratings) for ADHD with comorbid ODD/CD compared to ADHD only and compared to ADHD + anxiety was found in an MTA study [27] supporting our results of an association between ADHD symptoms and externalizing disorders (ODD/CD but not with internalizing disorders (anxiety and affective disorders.

The association of age with better global functioning and less ADHD symptoms found in this study is interpreted as a result of parents seeking psychiatric help for younger children when they have more severe symptoms, compared to seeking help for older children also when they have milder symptoms.

The association between ASD and ADHD symptoms was not foreseen. We hypothesized more severe ADHD symptoms in individuals with comorbid ODD/CD, and this was confirmed. The co-occurrence of ASD and ADHD involves a complex pattern and quality of symptoms, and impaired attention and impulsivity may in some cases originate in ASD rather than in ADHD [28]. It may be better to explore these issues through more dimension-specific research. Such studies have shown a strong association between hyperactivity/impulsivity and repetitive/restricted behaviors in ASD [29, 30]. A study of children with concurrent ADHD and ASD showed a significant association of greater psychiatric comorbidity with ADHD severity, but no association with ASD severity [31].

There is an intricate interplay between ADHD symptoms and symptoms from comorbid diagnoses. As an example a depression might influence the core symptoms of ADHD and worsen inattention as compared to when the depressive symptoms are not concurrent. Other diagnoses can add symptoms beyond the core symptoms of ADHD. For example ASD can cause more severe global functional impairment. The results of this study provide information on comorbid diagnoses and level of functional impairment and associations to ADHD symptoms, but the results do not reveal the mechanisms that may underlie the differences in associations among the disorders.

The estimated world prevalence in children and adolescents of any anxiety disorder is 6.5%, any depressive disorder 2.6%, and any disruptive disorder 5.7% [32]. These estimates are near the total prevalence of these diagnoses in our sample, although we expected the frequencies to be higher, given that a diagnosis of ADHD increases the odds of having an additional psychiatric diagnosis. The frequencies for affective and anxiety disorders, ODD/CD, and ASD in our sample are very similar to those found for younger Swedish children with ADHD by Giacobini et al. [7]. Their large study was based on mandatory registries of the ADHD population in Sweden. The most common comorbid disorder among younger ADHD patients with data from 2006 to 2011 in the Giacobini study, [7], was, as in our sample, ASD, even though the rate of ASD is larger in our study, which uses data from 2016 to 2017. Their study also showed an increasing rate of ADHD over time. Similarly, the prevalence of ASD in Sweden is increasing [33]. Between 2001 and 2011, there was an almost 3.5-fold increase of ASD diagnoses among children aged 2–17 years. The rate of diagnosed concurrent ADHD and ASD is also increasing, implied by the study by Giaconi et al. [7] with a higher prevalence in the young ADHD population, as confirmed by this study. Studies on co-occurring ADHD and ASD are limited, since the DSM-IV specifies ASD as an exclusion criterion for ADHD, although there are reports of a large overlap. An example is [34], who found a large overlap between ADHD and ASD, with 20–50% of children with ADHD meeting the criteria for ASD and 30–80% of children with ASD meeting ADHD criteria. A review by Gargaro and colleagues [35] reported a prevalence of ADHD with ASD of between 14 and 78%, and a recent meta-analysis estimated the pooled prevalence of concurrent ADHD and ASD to be 28% [36]. A genetic overlap between clinically ascertained ASD and ADHD diagnoses has been suggested [37]. The high frequency of registered ASD in combination with a low frequency of registration of other psychiatric diagnoses in this study is probably influenced by diagnostic routines in many clinics in Sweden when diagnosing ADHD: they routinely screen for other neurodevelopmental disorders, but they do not have the same focus on other psychiatric disorders.

Concurrent ADHD and ASD as well as multiple comorbidities pose a challenge for finding the best treatment strategies. More complicated planning of pharmacological and non-pharmacological treatment and follow-up can be assumed. However, the recommendations for pharmacological treatment in children and adolescents with ADHD and comorbid ASD are in line with guidance for treatment in ADHD alone [38]. The clinical implications of the association between ADHD and ASD on the course of ADHD symptoms are one of several issues of research interest.

We were limited to the documentation in the registry, with no way to control the information other than checking for impossible values. The CGAS score is based on the clinician’s judgement, and we cannot exclude information bias to some degree.

The study was conducted in the context of clinical every day practice, and the generalizability of our results to the population of children and adolescents with ADHD diagnoses in Sweden is limited to the group who have contact with psychiatric services and consequently more severe problems. There can also be qualitative differences between those who were asked to participate in the registry and those who were missed. However, we have no reason to believe that there are any systematic differences between these groups. A more likely source of selection bias is differences between those who agreed to participate and those who declined: it is possible that those who agreed to participate did so in part because they had more severe problems than those who declined, thus representing a particular subgroup of all children and adolescents with ADHD.

Frequency and regression analyses included six common ADHD comorbid disorders, but the effects of other disorders were not assessed. Finally, the results are cross-sectional and we cannot assess causality.

Despite missing registrations, a strength of this study is the sample size. The included patients came from a range of units and there is a national coverage of ADHD patients, resulting in a fair representation of young patients with ADHD who are receiving psychiatric care.

## Conclusions

ADHD in combination with multiple disorders, ASD, ODD/CD, anxiety, affective and learning disorders in decreasing order, were associated to low level of global functioning compared to ADHD only. ADHD with multiple disorders, ASD, ODD/CD and tic disorders were associated with more ADHD symptoms. Gender was not associated to the level of functioning, but there was an association to severity of inattention symptoms with boys having more inattention problems. Due to the impact of coexisting psychiatric disorders on clinical measures, the individual comorbidity profile should be taken into account in treatment plans for ADHD.

## Data Availability

The dataset analyzed during the current study is not available. It belongs to BUSA registry and can only be accessed through a formal application to the registry.
